# Can Carpal Tunnel Syndrome be Appropriately Diagnosed in a Cold Hand?

**DOI:** 10.51894/001c.25941

**Published:** 2021-08-30

**Authors:** Michael T. Andary, Drew B. Parkhurst, Maurice R. Bernaiche, Jose S. Figueroa, Lata Kumaraswamy, Suzanne M. Manzi, Ryan A. O’Connor, Ingrid P. Parrington, Jim R. Sylvain

**Affiliations:** 1 Physical Medicine & Rehabilitation Michigan State University College of Osteopathic Medicine, Sparrow Hospital, McLaren Greater Lansing Hospital; 2 New England Sports, Orthopedics, Spine, and Rehabilitation; 3 Des Moines University Clinic; 4 Southwest Spine and Sports; 5 Performance Pain & Sports Medicine; 6 Walter Reed National Military Medical Center

**Keywords:** nerve conduction study, electromyogram, carpal tunnel syndrome, temperature

## Abstract

**INTRODUCTION:**

The diagnosis of carpal tunnel syndrome (CTS) with nerve conduction studies traditionally involves warming the hand to avoid misleading prolongation of distal latency (DL). Comparing the median nerve DL to the ulnar and radial nerves using the combined sensory index (CSI) has been reported to improve the accuracy of CTS diagnosis. During this study, the authors examined the effect of hand temperature on the CSI and diagnosis of CTS.

**METHODS:**

The authors conducted a prospective, controlled, cohort study with 20 asymptomatic control patients and 21 symptomatic patients with confirmed CTS. Symptomatic patients underwent nerve conduction studies with the CSI calculated under both cold and warm conditions.

**RESULTS:**

Control subjects with warm hands had an average CSI of 0.0 milliseconds (ms), and -0.3ms with cold hands. CTS subjects with warm hands had an average CSI of 3.2ms, and 3.7ms with cold hands. Although hand temperature was shown to slow sample latencies, differences calculated with the CSI did not misclassify any of the 41 sample subjects.

**CONCLUSIONS:**

During this study, cold temperature did not result in misclassification of either control patients or CTS patients when CSI was diagnostically used. Based on these results, peak latency comparisons in cold hands can be considered as diagnostically reliable as under standard hand temperature ranges for the diagnosis of CTS, with caution warranted in borderline cases. This diagnostic technique can save time for the patient, physician, and care team without compromising quality of care. Future larger sample blinded studies at multiple electrodiagnostic sites are indicated.

## INTRODUCTION

Although carpal tunnel syndrome (CTS) has been classically described by numbness, tingling and weakness in the first three digits of the hand, the condition is more formally defined as an impairment of the median nerve caused by mechanical compression and/or local ischemia as it travels through the carpal tunnel.^1^ Electrodiagnostic testing aids in quantifying the extent of median nerve injury. Nerve conduction studies (NCS) in CTS have demonstrated prolongation of the median nerve distal latency (DL) across the wrist in sensory and/or motor fibers.^1^

Physiologic factors (e.g., temperature, age, height, limb length) can affect electrodiagnostic test results.^2^ Temperature has been shown to alter nerve conduction DL, waveform morphology, nerve conduction velocity, and is considered a sentinel factor defining normal values in NCS.^2–6^ Ideally, NCS are performed with an extremity temperature of 33°C^7^ but can be lower 30-32°C.^8^ Cooler temperatures (<33°C) predictably result in prolonged DL, increased amplitude and decreased nerve conduction velocity due to delayed opening and closing of sodium channels during an action potential.^2^

DL prolongation occurs when sodium channels open slowly.^2^ Sodium channels also remain open longer, resulting in more current flow and increased amplitudes on NCS.^2^ When electrodiagnostic testing reveals a large amplitude waveform with increased duration and slow conduction velocity, a cooling effect should be considered.^2^

In a warm extremity, skin surface temperature is 1-2°C warmer than near nerve temperature, and in a cool extremity, skin surface is typically colder than near nerve temperature.^9^ Whether or not accurate near nerve temperature measurements can be approximated by skin surface, subcutaneous or intramuscular temperature has been previously investigated.^4^

Studies have demonstrated a reliable relationship between intramuscular, subcutaneous and skin surface temperatures to allow electromyographers to utilize skin temperature alone.^5^ A temperature gradient in limbs has been demonstrated: warmer temperatures proximally and cooler temperatures distally.^4^ DeJesus et al. examined the effect of warm and cool skin surface temperatures on near nerve temperatures and found near nerve temperature changes ranging from 0-1.7°C, noting cooler skin surfaces had warmer near nerve temperatures and vice versa.^5^

This suggested to the authors that there would be cases in which the skin temperature could be acceptably warm, but the nerve still cold and vice versa. Occasionally, despite clinicians’ best efforts, some patients limbs are unable to be warmed to optimal temperatures for various reasons ranging from reduced muscle mass or decreased arterial blood flow.

When performing electrodiagnostic studies, utilization of a single test has been shown to possibly lead to false-positive results, in turn leading to unnecessary procedures.^10,11^ Sensitivity and specificity of carpal tunnel diagnosis can be increased with the combined sensory index (CSI), which demonstrates the prolongation of median nerve DL in relation to the ulnar and radial nerves.^1,10^ The CSI is the sum of DL differences in 1^st^ digit [thumbdiff], 4^th^ digit [ringdiff] and palm-to-wrist [palmdiff] (Table 1).^10^ If a calculated DL was negative, this was subtracted from the sum. When the CSI is ≥ 1.2, CTS diagnosis has a reported 82% sensitivity and 100% specificity.^11^

**Table 1.** Studies Performed^1^

SNCS with Ring Electrodes	SNCS with Bar Electrodes
D1 M: Antidromic distal sensory latency recorded on Digit 1 (D1) after stimulation of the median nerve 10 cm proximal to the active electrode D1 R: Antidromic distal sensory latency recorded on Digit 1 (D1) after stimulation of the radial nerve 10 cm proximal to the active electrode D2 M: Standard antidromic sensory latencies recorded on Digit 2 (D2) after stimulation 14 cm and 6 cm (mid palm) proximal to the active electrode D5 U: Standard antidromic sensory latencies recorded on Digit 5 (D5) after stimulation 14 cm and 6 cm proximal to the active electro D4 M: Antidromic distal sensory latency recorded on Digit 4 (D4) after stimulation of the median nerve 14 cm proximal to the active electrode D4 U: Antidromic distal sensory latency recorded on D4 after stimulation of the ulnar nerve 14 cm proximal to the active electrode	MP M: Orthodromic mixed nerve latency recorded at the wrist after stimulation of the median nerve in the palm 8 cm distally between the second and third metacarpals MP U: Orthodromic mixed nerve latency recorded at the wrist after stimulation of the ulnar nerve in the palm 8 cm distally between the fourth and fifth metacarcarpals

Key: SNCS: Sensory nerve conduction study, M: Median, R: Radial, U: Ulnar, MP Mixed Palm. The above tests were completed as documented above due to convention of the lab at the time of testing.

Warm hand temperature has generally been considered necessary in the valid interpretation of NCS. There are multiple ways to warm a patient’s cool hand, including immersion in warm water and using a hot pack.^4^ Warming the extremity takes time and can be inefficient in clinical practice, causing prolonged patient waiting times and studies. Current literature has not demonstrated whether the same magnitude of slowing occurs in the median, ulnar and radial nerves in cold conditions or how temperature affects a normal versus diseased median nerve.^12^

If latency differences (as opposed to absolute latencies) remain stable despite temperature changes, the diagnosis of CTS would be valid in either a cold or hot hand, saving the time and expense of temperature monitoring and hand warming.

### Study Objectives

By testing the effect of temperature on the DL of sensory median, ulnar, and radial nerves and its impact on CTS diagnosis, this study had three objectives:

1. To determine temperature effect on median sensory DL comparison techniques on normal subjects.
2. To determine temperature effect on median sensory DL comparison techniques in patients with diagnosed CTS.
3. To determine temperature effect on the electrodiagnosis of CTS using median sensory DL comparison techniques.

## METHODS

Full campus-based Institutional review board approval was obtained for the study design and all subjects were provided informed consent. Two groups of subjects were prospectively studied from 1998-2008 at the electrodiagnostics lab at McLaren Greater Lansing in Lansing, MI. All tests were performed at one site but performed by eight of the co-authored physicians under the supervision of Dr. Michael Andary. The control group consisted of 20 asymptomatic adults without evidence of peripheral neuropathy or symptoms consistent with CTS.

The control group was comprised of a convenience sample of people without nocturnal dysesthesias more than once per month, or signs or symptoms of polyneuropathy, or unexplained hand pain, diabetes, or other conditions that could cause polyneuropathy. Exclusion criteria for the control group included nocturnal numbness or paresthesias in the upper or lower extremities more than one time per month.^1^

The CTS group consisted of 21 adults referred to an academic physiatry clinic for electrodiagnostic testing. Inclusion criteria required NCS abnormalities in at least two of four different comparison tests (defined in Table 1 to demonstrate two tests that clearly agree) calculated from the sensory nerve action potentials (SNAPs) studied, or with CSI ≥ 1.2ms. At that time, the authors offered patients the opportunity to enroll in the study without reimbursement. Approximately 50% of eligible patients declined and the previously ordered electrodiagnostic evaluation was completed without additional study testing.

The authors believed that the additional testing was clinically justified because patients’ temperatures were between 30 and 33°C and additional testing with warming and cooling may have changed their diagnosis. One of the CTS group subjects was excluded for not meeting diagnostic criteria, leaving a final CTS group of 20 subjects. Exclusion criteria for this group were absent SNAP, or small SNAP amplitude of less than 7μV or evidence of any form of neuropathy other than CTS.

### Procedure

This prospective, controlled, unblinded study was conducted in the electrodiagnostic laboratory of a university affiliated regional medical center. Standard patient histories were taken followed during focused physical examinations. Room temperature was maintained at 23.9°C. Hand temperatures were recorded with CE Infrared Thermometer TN153F at multiple sites: Second digit proximal interphalangeal {PIP} joint (D2), fifth digit PIP joint (D5), third metacarpal head (MC) and carpal tunnel approximately 2cm distal to the distal wrist crease (CT).^1^

The performing physician then maintained skin temperature of all sites in the acceptable range of 30-33°C and was given 15 minutes to complete testing. Using an XLTEC NeuroMax 1002, subjects underwent a standard electrodiagnostic carpal tunnel screen.^1^ Enrolled patients then underwent repeat electrodiagnostic testing under two separate temperature conditions, “hot” and “cold”, as defined below. Control subjects did not have the evaluation between 30 and 33°C, only the evaluation of the two extreme cases.

**Hot:** Skin was warmed with one or two hydrocollator packs placed on the forearm, wrist and hand. When skin temperature reached ≥33°C, active, reference, and ground electrodes were placed on the hand and the same CTS screen was repeated. At no time were test data acquired from an arm with skin temperature <33°C at any of the four locations. If the temperature did drop, the extremity was rewarmed and the procedure repeated, this was infrequent and occurred approximately in 10% of patients.

**Cold:** Immediately following the hot portion of the study, each subject’s forearm and hand were submersed in a tub of cool running water maintained between 25-28°C until a skin temperature of <30°C was recorded for a period of five minutes. The cold extremity was dried before active, reference, and ground electrodes were placed and the CTS screen was repeated. At no time were data collected from a limb with a skin temperature >30°C. If the temperature rose, the limb was re-cooled and the procedure repeated.

Calculated Latencies:

Thumbdiff=D1M-D1R

D2,5=D2M-D5U

Ringdiff=D4M-D4U

Palmdiff=MPM-MPU

### CTS Diagnostic Criteria

Two scales were used for diagnosis of CTS, using abnormal comparison studies and using the CSI. These criteria were used in our laboratory based on our own studies,^1^ and corroborated with other studies in the literature.^10,11^

1. Subjects with two or more of these calculated DL values:
  Thumbdiff ≥ 0.4ms
  D2,5 ≥ 0.4ms
  Ringdiff ≥ 0.5ms
  Palmdiff ≥ 0.4ms
2. CSI: If thumbdiff +ringdiff + palmdiff ≥ 1.2, we considered this abnormal for the electrodiagnosis of CTS (positive and negative

### Data Analyses

Since NCS data are non-parametrically distributed, using simple means and standard deviations may have been misleading. Therefore, the authors attempted conversions to make them parametric including square root, 1/square root, squared, 1/squared, cubed, 1/cubed conversions, however, this was unsuccessful. Due to the size of their sample, the authors examined individuals at either post-testing extreme, with special attention to sample patients who may have changed in classification from normal to CTS abnormal or vice versa.

## RESULTS

Unsurprisingly, we found DL in cold hands considerably slower than the warm hands, and the statistics were not parametric. These findings were expected, not noteworthy, and therefore not further discussed. However, NCS performed on cold hands affected the differences in DL in a variable and not easily predictable manner. This finding is further reported below.

### Thumbdiff

**Control Subgroup**: Thumbdiff studies in control subjects resulted in a result of <0.4ms in 19 (95%) subjects under both the hot and cold conditions. Range in hot condition was -0.3 to 0.3 ms and range in cold condition was -0.8 to 0.5 ms.

**CTS Subgroup**: In the hot temperature condition, 19 (95%) CTS subjects had an abnormal thumbdiff (≥0.4ms), and range 0.4-2.6 ms. Eighteen (90%) subjects in the CTS group had abnormal thumbdiff in the cold condition. Range in the cold condition was 0.1 to 2.3 ms.

**Figure 1.**
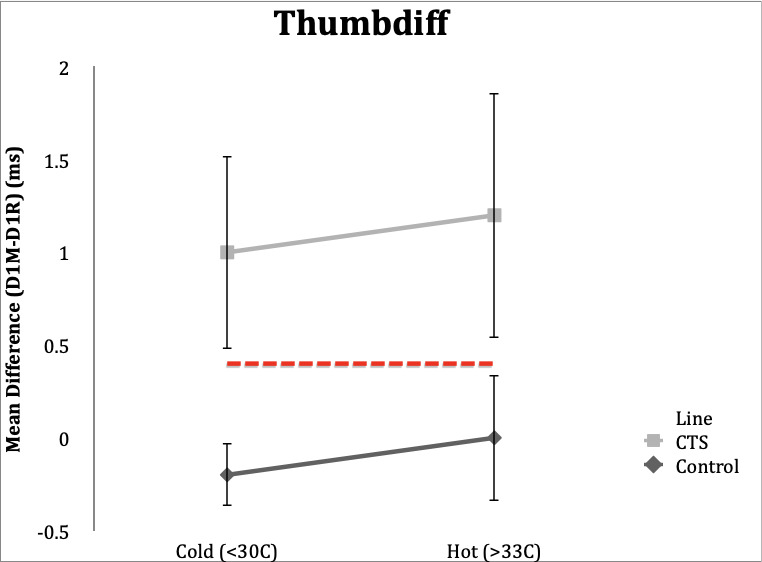
Mean differences in DL comparing median and radial nerves at the thumb in control and CTS subjects in hot and cold temperature conditions. Error bars demonstrate one standard deviation from mean. Dotted line delineates upper limit of normal for Thumbdiff (0.4ms).

### D2,D5

**Control Subgroup**: In hot conditions, 18/20 (90%) controls had normal values and one of 20 (5%) had a value of 0.4ms, and range of -0.4 to 0.4 ms. In cold conditions, all subjects were within the normal value of <0.4ms, however three (15%) were in the upper limit of normal (0.3ms) and could be considered borderline. Range was -0.4 to 0.3 ms.

**CTS Subgroup**: Unlike the other studies, temperature in the D2, D5 study changed classification in five subjects (25%) when their hands were cold. Fifteen subjects (75%) retained abnormal differences in distal latencies in the hot condition, and 16 subjects (80%) had distal latencies ≥0.4ms. Variability was present in D2, D5 studies within CTS subjects.

Four subjects (20%) in the hot condition had DLs within normal limits, while in the cold condition these same subjects had abnormal latencies. Conversely, three subjects (15%) had normal latencies in the cold condition and had abnormal latencies in the hot condition. Range in the hot condition was 0.0 to 2.2 ms and range in the cold condition was 0.0 to 2.5 ms.

**Figure 2.**
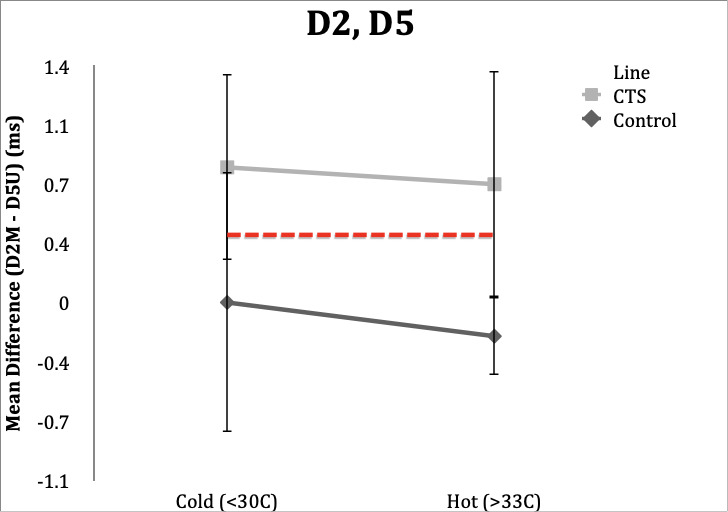
Mean differences in DL comparing index finger at the carpal tunnel and pinky finger at Guyon’s canal in control and CTS subjects in hot and cold temperature conditions. Error bars demonstrate one standard deviation from mean. Dotted line delineates upper limit of normal for D2,D5 (0.4ms).

### Ringdiff

**Control Subgroup**: In the hot condition, all control subjects (100%) had normal values (<0.5ms) for ringdiff, and range of -0.3 to 0.4 ms. When the studies were repeated in the cold condition, all but one subject (95%) had normal studies. Range was -0.5 to 0.5ms.

**CTS Subgroup**: Eighteen subjects (90%) in the CTS group had abnormal values (≥0.5ms) in both hot and cold conditions. Range in hot condition was 0.2-3.9 ms and range was -0.1 to 4.8 ms in the cold condition.

**Figure 3.**
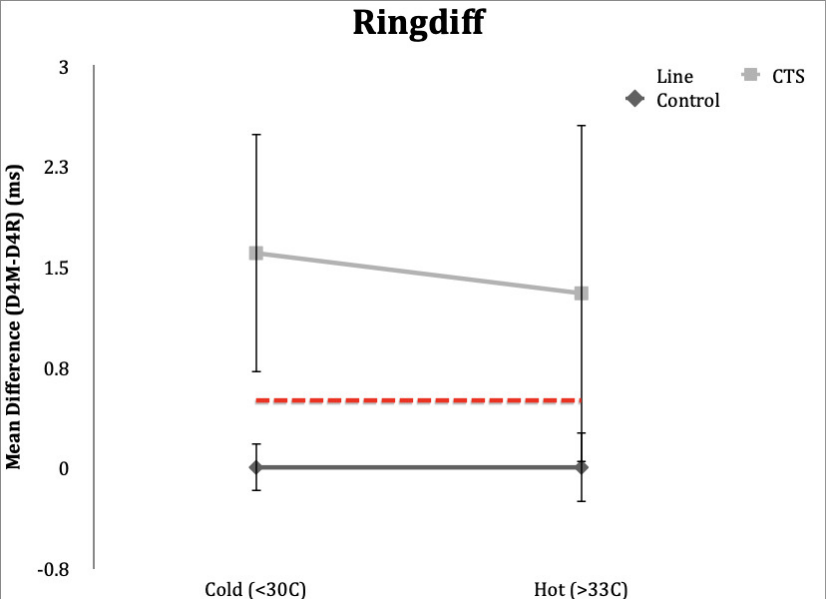
Mean differences in DL comparing median and ulnar nerves in the ring finger in control and CTS subjects in hot and cold temperature conditions. Error bars demonstrate one standard deviation from mean. Dotted line delineates lower limit of normal for Ringdiff (0.5ms).

### Palmdiff

**Control Subgroup**: All 20 subjects (100%) had normal values (<0.4ms) in the hot and cold conditions. Range in the hot condition was -0.4 to 0.3 ms and range in the cold condition was -0.4 to 0.3 ms.

**CTS Subgroup**: In the hot condition, 18 subjects (90%) had abnormal (≥0.4ms) latencies and 2 subjects (10%) had latencies within normal limits with a range of 0.2 to 1.4 ms. In the cold condition, all 20 CTS subjects (100%) had abnormal latency values. Range was 0.4 to 2.7 ms.

**Figure 4.**
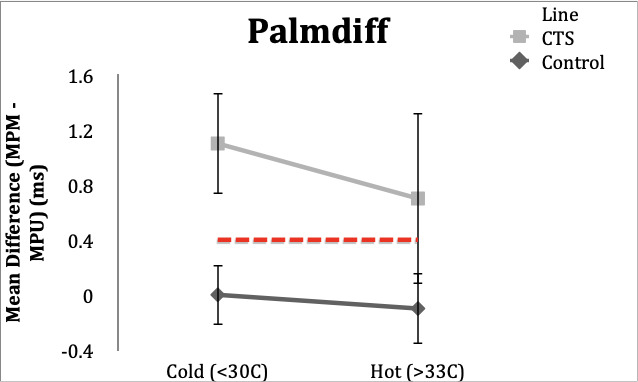
Mean differences in DL comparing mixed palm to wrist in control and CTS subjects in hot and cold temperature conditions. Error bars demonstrate one standard deviation from mean. Dotted line delineates lower limit of normal for Palmdiff (0.4ms).

### CSI

**Control Subgroup**: All 20 control subjects (100%) had CSI values of <1.2ms in the cold and hot conditions. In cold conditions, one of the 20 subjects (5%) had a CSI in the upper limit of normal. Range in hot conditions were -0.6 to 0.9 ms, and range in the cold condition was -1.3 to 1.1 ms.

**CTS Subgroup**: CSI was abnormal (≥1.2ms) in 19 subjects (95%) in the hot condition and 18 subjects (90%) in the cold condition. Range in the hot condition was 0.9 to 7.0 ms and range in the cold condition was 1.1 to 8.6 ms.

**Figure 5.**
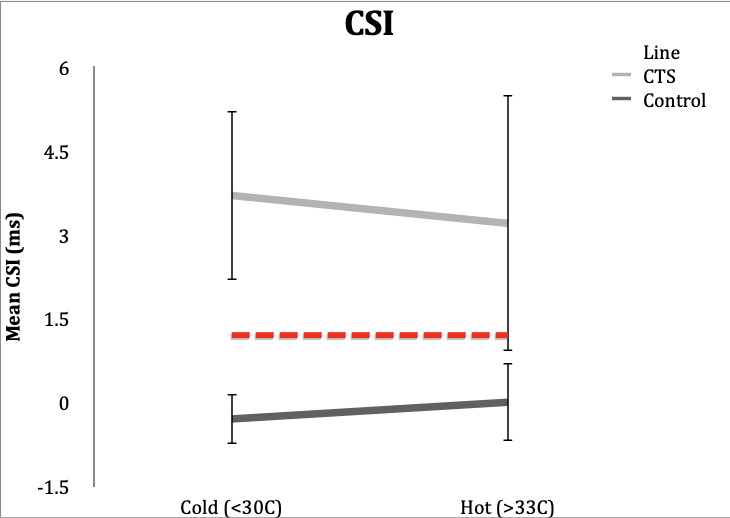
Mean differences in DL of the combined sensory index in control and CTS subjects in hot and cold temperature conditions. Error bars demonstrate one standard deviation from mean. Dotted line delineates lower limit of normal for CSI (1.2).

NCS performed in cold hands demonstrated a variable and not easily predicted effect on sensory latency differences. Using the CSI for diagnosis, cooling of the hand did not cause misclassification of subjects with or without CTS. In normal subjects with warm hands the average CSI was 0.0 ms. In normal subjects with cold hands, the average CSI was -0.3 ms, suggesting that cold hands made normal hands less likely to be misclassified.

In CTS subjects with warm hands, the average CSI was 3.2 ms, while CTS subjects with cold hands had an average CSI of 3.7 ms. In this situation, cold hands made the person more abnormal. In the study, four subjects (20%) had borderline results, however the changes in temperature did not cause misclassification. Thus, changing the temperature did not move individual borderline cases from normal to an abnormal status or vice versa.

In both normal participants and in those with CTS, evaluation of the cold hand did not result in a misclassification. In normal subjects, warming the hand resulted in a continued normal CSI, whereas in participants with CTS, warming the hand resulted in even more abnormal values. Warming the hand in borderline participants caused variable changes but did not change any individual patients from normal to abnormal status or vice versa .

## DISCUSSION

Nerve conduction studies performed in cold hands affect sensory latency differences in a variable and not easily predictable manner. However, temperature change occasionally misclassified a patient or a control when utilizing a single test.^1,11^ Using the CSI we did not find any cases of misclassification in our total sample of 40 patient cases.

The traditional requirement that the hand be warmed to >33˚C is logical when interpreting absolute DL and there is overwhelming evidence to support this.^3–6,8,12^ However, clinicians faced with patient with cold hands in busy practice settings may not always follow these guidelines to warm the hand.^13^ Although this problem has rarely been addressed in the literature, it is likely common in many laboratories and offices.

We cannot find evidence-based studies that have addressed the specific situation for CTS using comparison techniques.^6,8,9,11,12^ It is possible that patients with cold hands may still be reliably evaluated for CTS using comparison techniques. This study is the first apparent study that used data from patients with CTS and these commonly used comparison techniques in both warm and cold contexts.

Two responses to the electrodiagnostic workup of CTS in the cold hand have been previously described. In 2013, Preston et al. predicted DL adjustments corresponding to temperature changes: for each 1˚C drop in temperature, the DL is prolonged by 0.2 ms, however, they do not specifically discuss comparison techniques.^9^ A second earlier approach described by Robinson et al. utilized the CSI to normalize results and to decrease the effect of minor variables, such as, temperature.^11^ However, no data regarding temperature was presented.^11^

In 2010, Sandin et al. reported that warming the limb and measuring it again prior to electrodiagnostic testing is essential to reduce the likelihood of false positive results.^7^ Some electromyographers utilize conversion factors to correct for low skin temperatures. However, these conversion factors were acquired with healthy subjects and not in patients with diseased nerve.^7^ Research by Burnham et al. in 2009 suggested that warming the hand for 20 minutes increased the transcarpal tunnel conduction velocity and reduced waveform amplitude, resulting in misdiagnosis of normal and CTS, respectively.^8^ This can therefore generate potential error in cases that are borderline and can result in a possible misdiagnosis of carpal tunnel syndrome.^8^

### Study Limitations

First, a relatively small convenience sample of patients from a single physiatry clinic was enrolled by the authors. Successfully conducting these tests on patients under both warm and cold conditions was also quite difficult due to their limited time availability and expressed discomfort concerns. This led to a convenience sample of patients who were both willing to complete this study and we had the time to complete the extra NCS in both the hot and cold environments, possibly limiting the external generalizability of our results.

## CONCLUSIONS

Based on our study findings, the peak latency differences in cold hands can be as reliable as in standard temperature ranges for the diagnosis of CTS if CSI criteria are used. Hand temperature may have the least effect in changing diagnosis for subject who were clearly normal and those who clearly had CTS. Clinicians need to consider that borderline cases may still be misclassified in cold hands although we did not find it during this study. However, the technique described in this paper may still save time for patients, physicians, and other healthcare team members without compromising quality of patient care. Larger sample blinded studies at multiple electrodiagnostic sites are clearly warranted.

### Conflicts of Interest

None of the authors have any conflicts of interest to disclose.
